# Supplementary material for: Pairwise effects between lipid GWAS genes modulate lipid plasma levels and cellular uptake
Source: Nat Commun. 2021 Nov 5;12:6411. doi: 10.1038/s41467-021-26761-3 (PMC8571362; doi:10.1038/s41467-021-26761-3)
Supplement: Supplementary file 1 — Supplementary Information [file 41467_2021_26761_MOESM1_ESM.pdf]

Supplementary Information

Pairwise effects between lipid GWAS genes modulate lipid plasma levels and cellular uptake

Magdalena Zimoń[$], Yunfeng Huang[$], Anthi Trasta[$], Aliaksandr Halavatyi, Jimmy Liu, Chia-Yen Chen, Peter Blattmann, Bernd Klaus, Chris Whelan, David Sexton, Sally John, Ellen Tsai, Wolfgang Huber, Rainer Pepperkok*[#], Heiko Runz*[#]

[$] These authors contributed equally: Magdalena Zimoń, Yunfeng Huang, Anthi Trasta
[#] These authors jointly supervised this work: Rainer Pepperkok and Heiko Runz
* Correspondence to heiko.runz@gmail.com or pepperko@embl.de

Contents

Supplementary Figures

Supplementary Fig. 1: PTV-burden analysis results for the remaining lipid traits.
Supplementary Fig. 2: Cellprofiler pipeline for quantitative image analysis of LDL-uptake assay
Supplementary Fig. 3: Boxplot representation of the 20 gene interactions that were replicated with liquid phase transfection.
Supplementary Fig. 4: Subcellular localization of LDLR upon knockdown of interacting genes.
Supplementary Fig. 5: Plasma membrane expression of LDLR is unaffected by *LDLRAP1* knockdown
Supplementary Fig. 6: No effect of *TOMM40* knockdown on *APOE* expression levels.

Supplementary Tables

Supplementary Table 1: List of primers used for RT qPCR.

Supplementary Fig.1

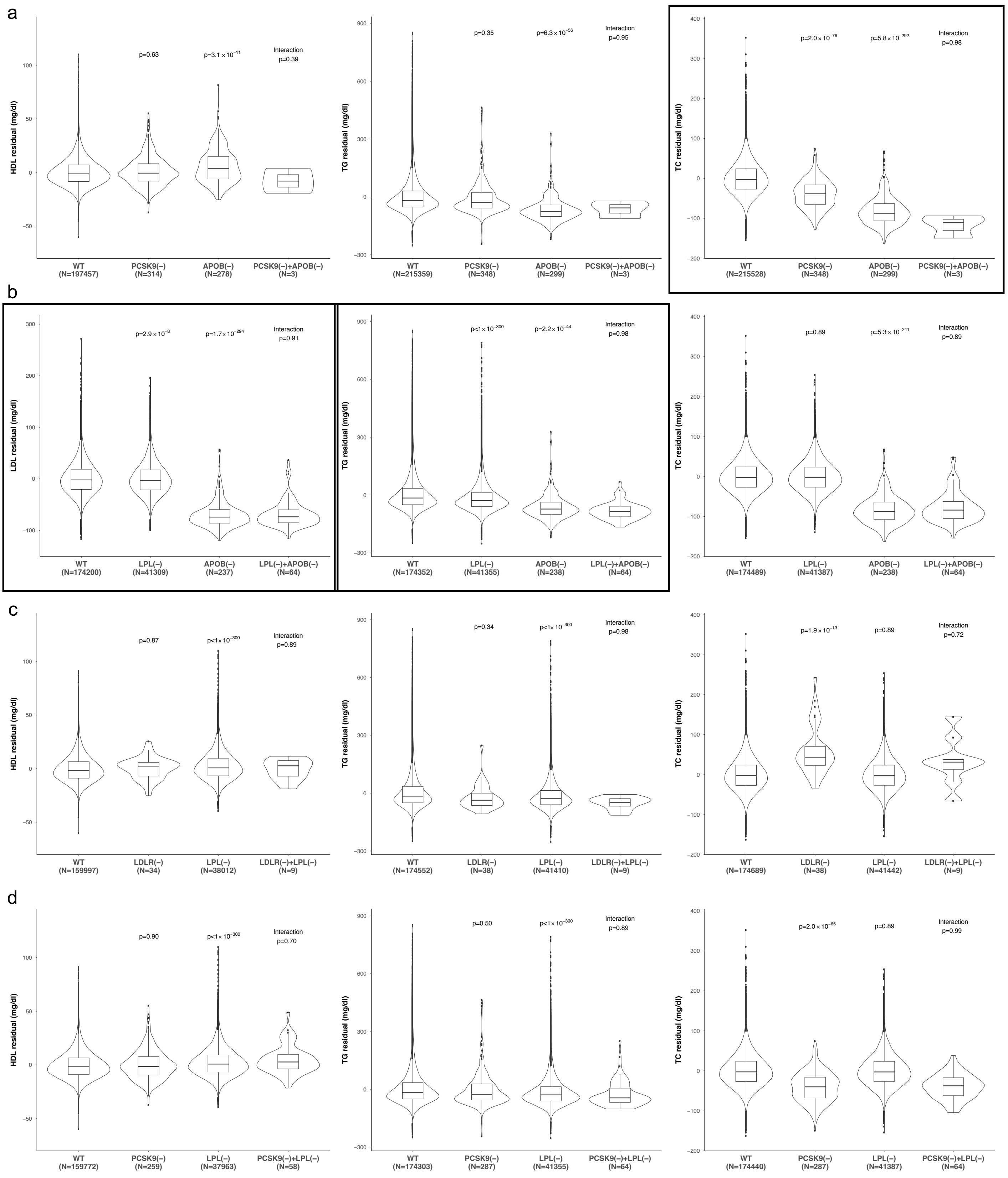

Supplementary Fig.1. PTV- burden analysis results for the remaining lipid traits. Gene-based PTV burden analyses from 302,331 exomes identified four gene pairs with additive effects. On this figure shown are effects on plasma levels in carriers of PTVs in **a** APOB and PCSK9, **b** APOB and LPL, **c** LDLR and LPL, **d** PCSK9 and LPL for the remaining lipid traits tested yet not depicted on Figure 1. Minimum and maximum values of residualized lipid measures as well as the data distribution were shown by violin plots, box in the middle indicates 25 and 75 percentiles with a horizontal black line indicating the median. Shown are p-values for single-gene effects as well as the interaction effect derived from robust linear model fit and corrected for multiple comparisons with FDR method (see Methods). n- number of carriers, (-) predicted loss-of-function due to PTVs. Significant results are those for which FDR corrected p-value is <0.005.

Supplementary Fig. 2

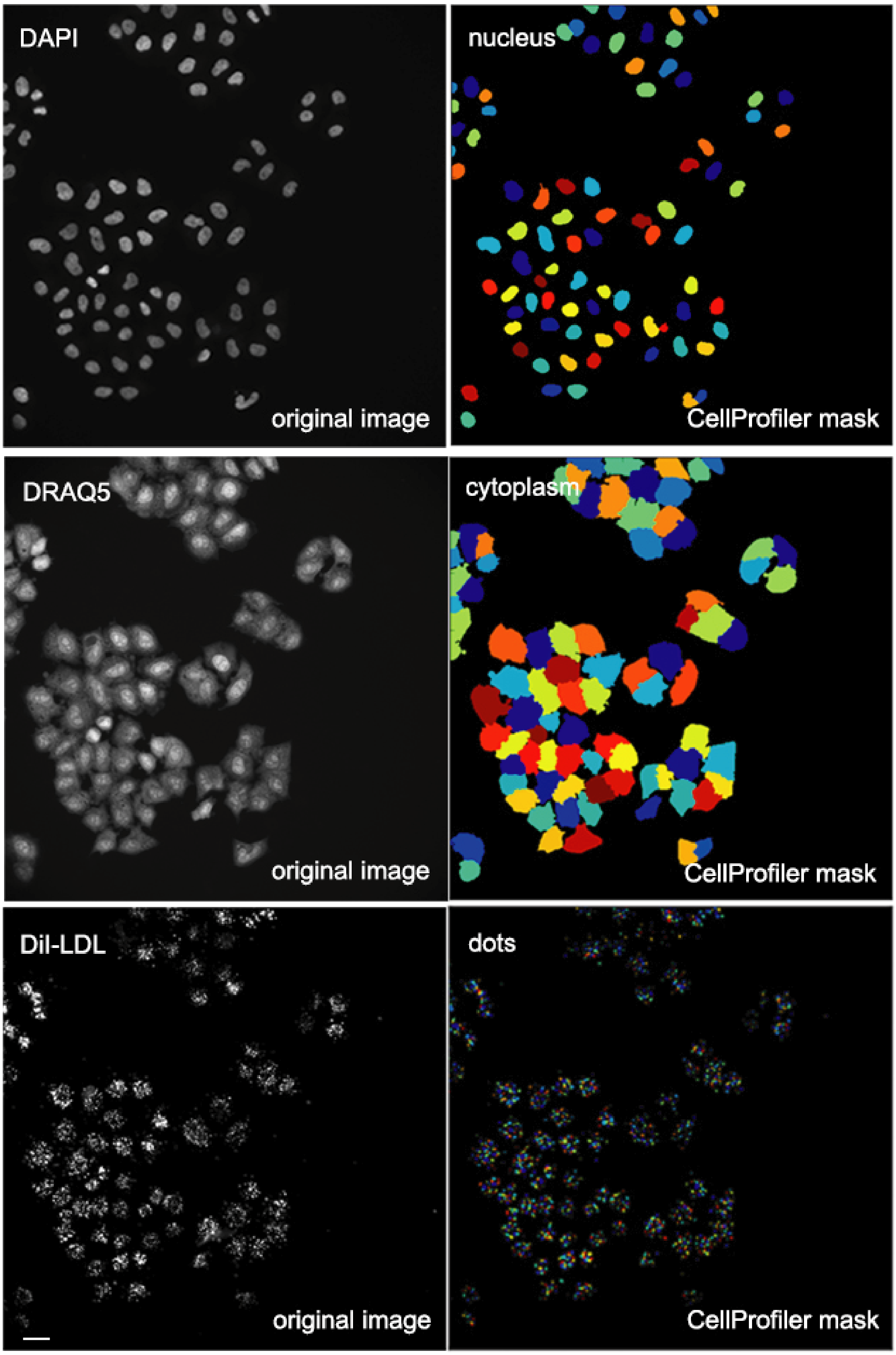

Supplementary Fig. 2. Cellprofiler pipeline for quantitative image analysis of LDL-uptake assay. Shown are representative images from the LDL-uptake assay (left column) acquired with the Scan^R software of the Olympus widefield microscope with the 20x objective and the segmentation of cellular structures (right column) performed through a CellProfiler pipeline. Scale bar 20 μm.

Supplementary Fig. 3

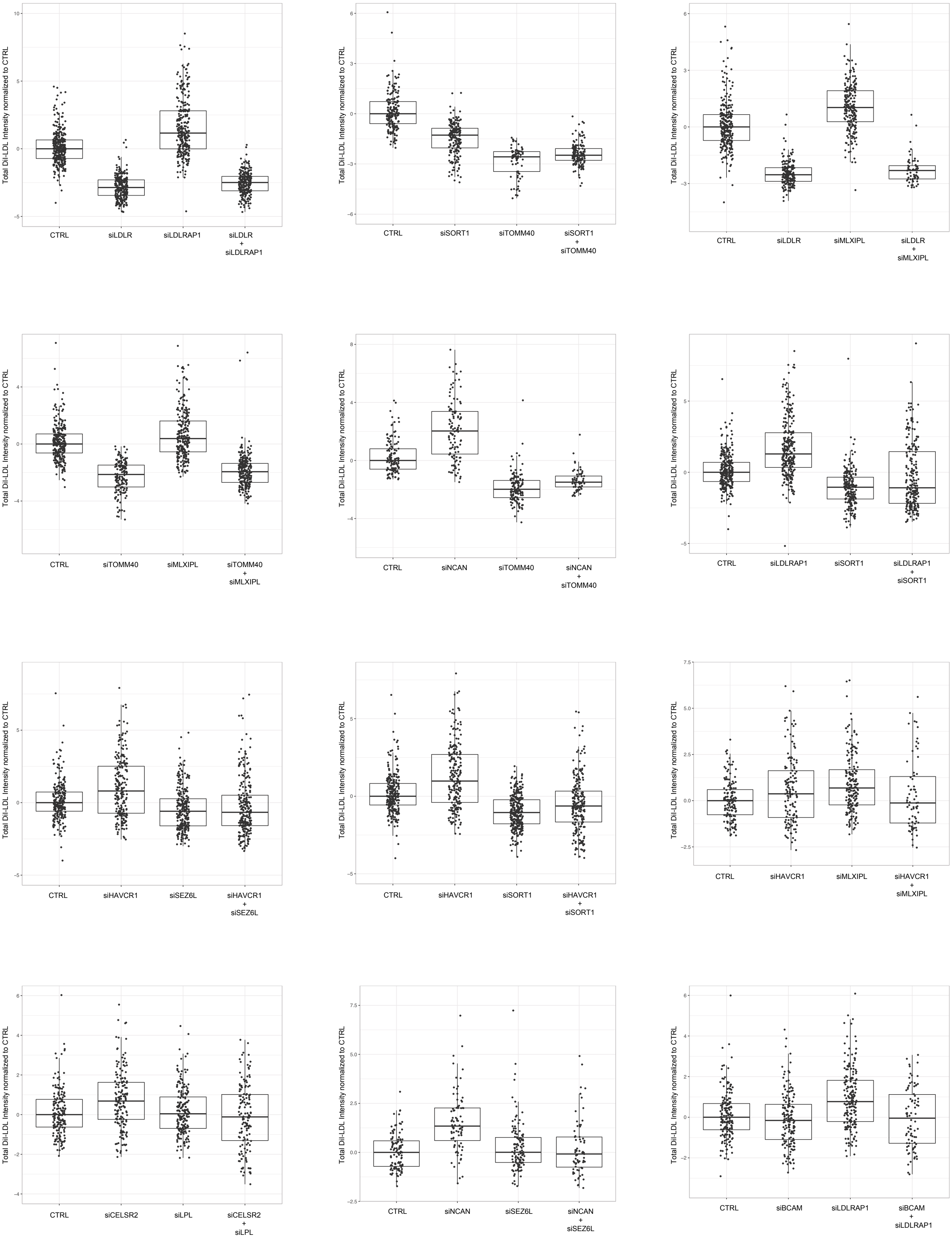

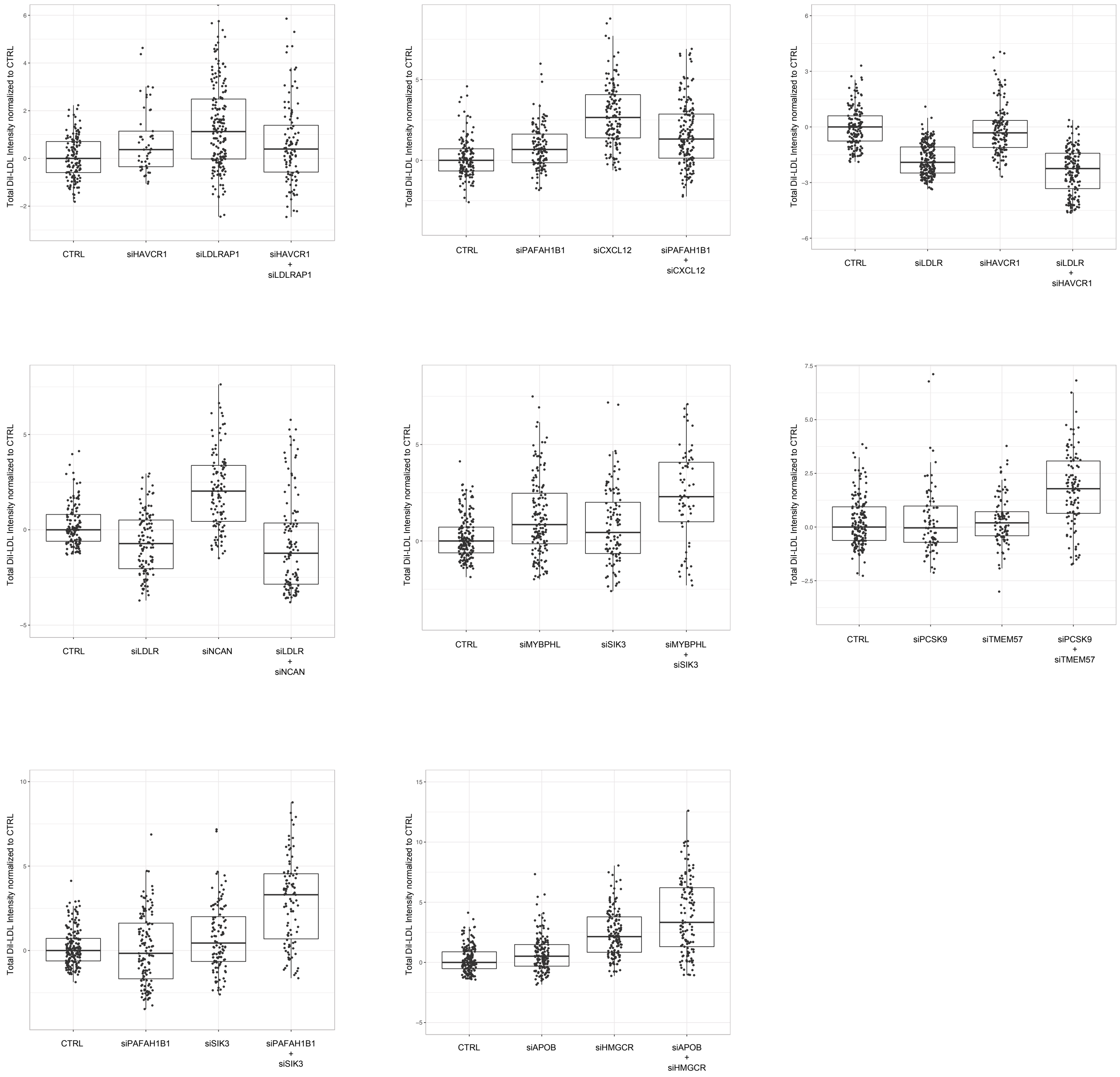

Supplementary Fig. 3. Boxplot representation of the 20 gene interactions that were replicated with liquid phase transfection.

Shown are the 20 gene interactions (GIs) that were validated with liquid-phase transfection.

Dots on boxplots represent robust Z-score values calculated for integrated DiI fluorescence intensities of cells averaged per image (Methods), showing the data distribution including minimum and maximum values. Boxplots represent values between 25th and 75th percentile, horizontal black line indicates median robust-Z-score calculated from quality controlled images - exact numbers of images are detailed in Supplementary Data 11. All images are originating from minimum n=3 independent biological replicas. Whiskers indicate largest value within 1.5 times interquartile range above 75th percentile. All validated GIs have $p_{(FDR)}<0.01$ that was derived from robust linear model fit and corrected for multiple comparisons with FDR method (see Methods).

Supplementary Fig. 4

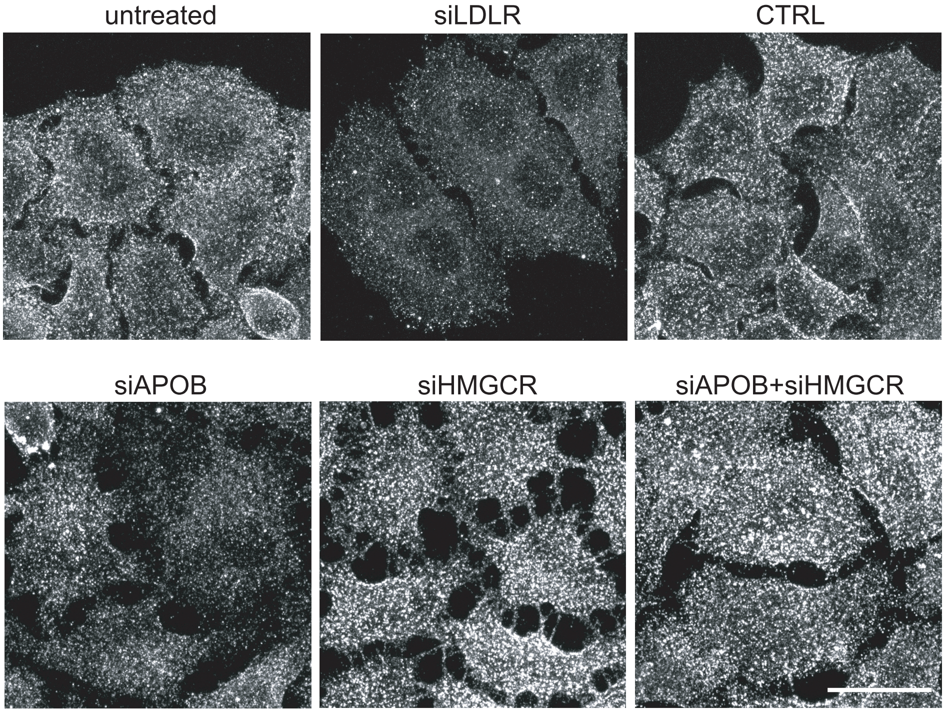

Supplementary Fig. 4. Subcellular localization of LDLR upon knockdown of interacting genes. HeLa Kyoto cells were transfected with siRNAs targeting products of indicated genes and stained with antibody against LDLR. Cells were grown under sterol-depleted conditions (see Materials and Methods). Shown are maximal projections of confocal stacks of representative cells from single experiment. Scale bar=20 μm.

Supplementary Fig. 5

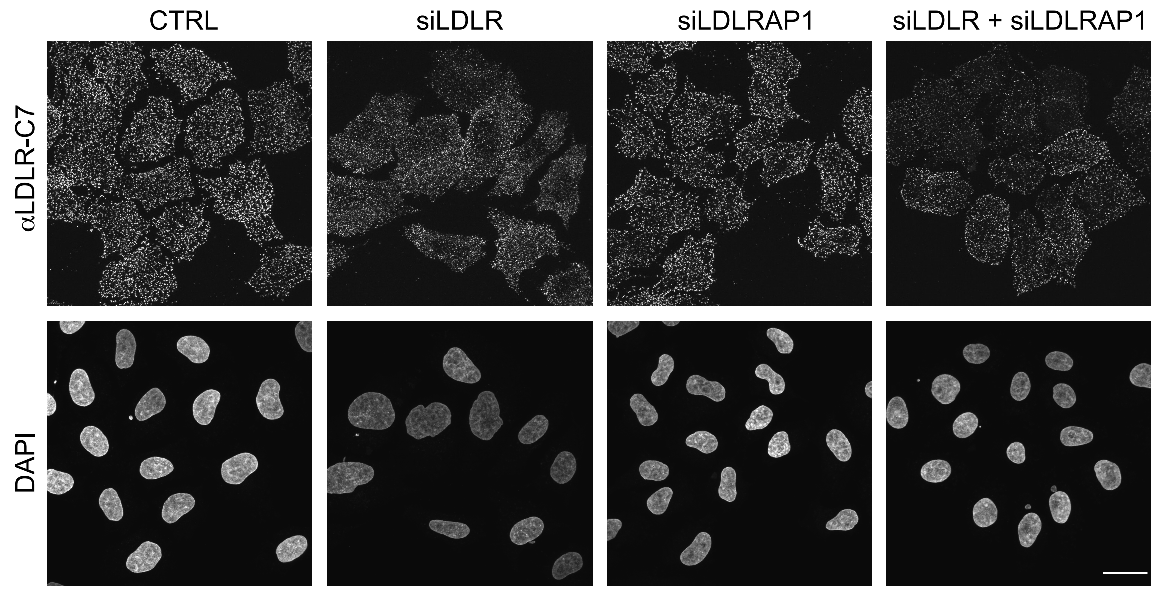

Supplementary Fig. 5. Plasma membrane expression of LDLR is unaffected by *LDLRAP1* knockdown.
After 48 hour knockdown of *LDLR* and *LDLRAP1*, LDLR was detected with anti-LDLR-C7 antibody recognising extracellular part of the receptor. Images show representative cells from single experiment. Scale bar = 10 μm.

Supplementary Fig. 6

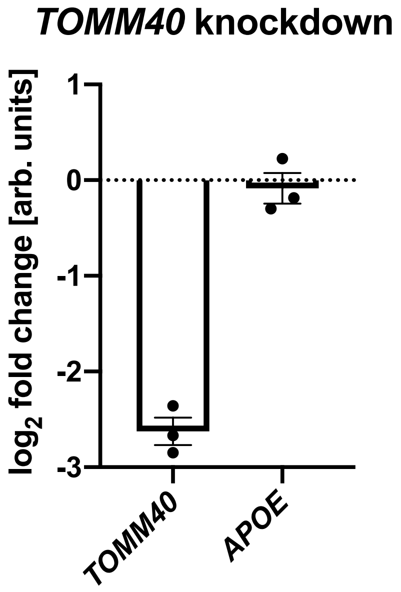

Supplementary Fig. 6. No effect of *TOMM40* knockdown on *APOE* expression levels. Shown are mRNA levels of either *TOMM40* or *APOE* in HeLa Kyoto cells after 48 h knockdown of *TOMM40* after normalization to the control siRNA. The target gene mRNA levels were normalized to the housekeeping gene, *GAPDH*. The error bars represent the standard error of the mean (n=3).

Supplementary Table 1

Gene	Forward primer 5´- 3’	Reverse primer 5´ - 3´
APOE	CTGCTCAGCTCCCAGGTC	TTGTTCCTCCAGTTCCGATT
GAPDH	CATGAGAAGTATGACAACAGCCT	AGTCCTTCCACGATACCAAAGT
TOMM40	ATTCAGATGGAGGGTGTCAAG	ATTGTGCTGAGGGCTACTGT

Supplementary Table 1. List of primers used for RT qPCR. Primers used for the analysis of the expression changes of *APOE* and *TOMM40* in HeLa Kyoto cells upon knockdown of *TOMM40*.
