# Supplementary material for: Pairwise effects between lipid GWAS genes modulate lipid plasma levels and cellular uptake
Source: Nat Commun. 2021 Nov 5;12:6411. doi: 10.1038/s41467-021-26761-3 (PMC8571362; doi:10.1038/s41467-021-26761-3)
Supplement: Supplementary file 3 — Description of Supplementary Dataset [file 41467_2021_26761_MOESM3_ESM.pdf]

Description of additional Supplementary Files

File Name: Supplementary Data 1.
Description: GWAS support for 30 lipid/CAD candidate genes analyzed in this study

File Name: Supplementary Data 2.
Description: A priori evidence for lipid/CAD-relevant biological functions of the candidate lipid genes under study

File Name: Supplementary Data 3.
Description: PTVs identified in the 30 lipid/CAD GWAS genes through exome sequencing of 302,331 UK Biobank participants

File Name: Supplementary Data 4.
Description: Single-gene PTV-burden association results for the 30 lipid/CAD GWAS genes with four lipid traits

File Name: Supplementary Data 5.
Description: Pairwise gene-based PTV-PTV burden interaction analysis results in 240,970 UK Biobank participants

File Name: Supplementary Data 6.
Description: PTV-PTV interaction for LPL stratified by S447X

File Name: Supplementary Data 7.
Description: Pairwise lipid/CAD GWAS lead SNP-SNP interaction analysis results in the UK Biobank for the 28 loci analyzed in this study

File Name: Supplementary Data 8.
Description: Results for testable pairwise GWAS lead SNP-PTV burden interaction analysis

File Name: Supplementary Data 9.
Description: Results for modifier effects between PRS and PTV burden interaction analysis

File Name: Supplementary Data 10.
Description: Results and statistical analysis of primary coRNAi screen

File Name: Supplementary Data11.
Description: Replication of coRNAi screening results independently validate 20 gene pairs as showing non-additive effects on cellular LDL-uptake
