# Supplementary material for: Pairwise effects between lipid GWAS genes modulate lipid plasma levels and cellular uptake
Source: Nat Commun. 2021 Nov 5;12:6411. doi: 10.1038/s41467-021-26761-3 (PMC8571362; doi:10.1038/s41467-021-26761-3)
Supplement: Supplementary file 15 — Reporting Summary [file 41467_2021_26761_MOESM15_ESM.pdf]

nature portfolio

Corresponding author(s): Heiko Runz and Rainer Pepperkok

Last updated by author(s): Aug 19, 2021

Reporting Summary

Nature Portfolio wishes to improve the reproducibility of the work that we publish. This form provides structure for consistency and transparency in reporting. For further information on Nature Portfolio policies, see our [Editorial Policies] and the [Editorial Policy Checklist].

Statistics

For all statistical analyses, confirm that the following items are present in the figure legend, table legend, main text, or Methods section.

n/a	Confirmed	
☐	☒	The exact sample size (*n*) for each experimental group/condition, given as a discrete number and unit of measurement
☐	☒	A statement on whether measurements were taken from distinct samples or whether the same sample was measured repeatedly
☐	☒	The statistical test(s) used AND whether they are one- or two-sided<br>*Only common tests should be described solely by name; describe more complex techniques in the Methods section.*
☐	☒	A description of all covariates tested
☐	☒	A description of any assumptions or corrections, such as tests of normality and adjustment for multiple comparisons
☐	☒	A full description of the statistical parameters including central tendency (e.g. means) or other basic estimates (e.g. regression coefficient) AND variation (e.g. standard deviation) or associated estimates of uncertainty (e.g. confidence intervals)
☐	☒	For null hypothesis testing, the test statistic (e.g. *F*, *t*, *r*) with confidence intervals, effect sizes, degrees of freedom and *P* value noted<br>*Give P values as exact values whenever suitable.*
☐	☒	For Bayesian analysis, information on the choice of priors and Markov chain Monte Carlo settings
☐	☒	For hierarchical and complex designs, identification of the appropriate level for tests and full reporting of outcomes
☐	☒	Estimates of effect sizes (e.g. Cohen's *d*, Pearson's *r*), indicating how they were calculated

Our web collection on statistics for biologists contains articles on many of the points above.

Software and code

Policy information about availability of computer code

Data collection	* Genetic data collection: detailed in Methods and available through UK Biobank website (http://www.ukbiobank.ac.uk)<br>* siRNA screen data: Image acquisition was performed using an Olympus IX81 automated microscope with Scan^R software<br>* RT qPCR data: StepOne Software v2.3
Data analysis	* RNAi screen data were analyzed with CellProfiler (v2.1.1. http://www.cellprofiler.org), HTM explorer (https://github.com/embl-cba/shinyHTM); R-script and HTM explorer exacuted using R 4.0.0 GUI 1.71 and RStudio (1.2.5042); FiJi v2.1.0/1.53c,<br>* Genetic data were analyzed with Variant Effect Predictor v96 (McLaren et al., 2016); LOFTEE plugin (Karczewski et al., 2020); PRS-CS (Ge et al., 2019); PhenoScanner platform(http://www.phenoscanner.medschl.cam.ac.uk/); 2013 Global Lipid Genetics Consortium GWAS1 (http://csg.sph.umich.edu/willer/public/lipids2013/);<br>* CellProfiler pipeline and R-scripts for GI determination and significance testing, plotting for genetic data and coRNAi screen - with detailed description of the code are publicly available at: https://git.embl.de/grp-almf/genetic-interactions-screen-lipid-levels

For manuscripts utilizing custom algorithms or software that are central to the research but not yet described in published literature, software must be made available to editors and reviewers. We strongly encourage code deposition in a community repository (e.g. GitHub). See the Nature Portfolio guidelines for submitting code & software for further information.

March 2021

Data

Policy information about availability of data

All manuscripts must include a data availability statement. This statement should provide the following information, where applicable:

- Accession codes, unique identifiers, or web links for publicly available datasets
- A description of any restrictions on data availability
- For clinical datasets or third party data, please ensure that the statement adheres to our policy

*Genetic data utilized in this study are publicly available to registered researchers through UK Biobank data-access protcol. Additional information about registration for access to the data are available at http://www.ukbiobank.ac.uk/register-apply/. Data for this study were obtained under Resource Application Number 26041.
*coRNAi screening data - all source imaging data for primary and validation screen along with confocal images are deposited at https://idr.openmicroscopy.org/about/. Cell Profiler pipeline and all R-codes required to fully recapitulate how results were generated from coRNAi-analyses in a step-by-step manner have been deposited at https://git.embl.de/grp-almf/genetic-interactions-screen-lipid-levels.
*Additional data that support findings of this study can be made available through contacting the corresponding author Heiko Runz (heiko.runz@gmail.com).

Field-specific reporting

Please select the one below that is the best fit for your research. If you are not sure, read the appropriate sections before making your selection.

☒ Life sciences ☐ Behavioural & social sciences ☐ Ecological, evolutionary & environmental sciences

For a reference copy of the document with all sections, see nature.com/documents/nr-reporting-summary-flat.pdf

Life sciences study design

All studies must disclose on these points even when the disclosure is negative.

Sample size	*This study utilizes whole-exome sequencing data from 302,331 participants of the UK Biobank as generated by the UK Biobank Exome Sequencing Consortium. Moreover, genotype data from a total of 387,033 UK Biobank participants was utilized. No sample size calculations for power were performed<br>*For siRNA screen - sample size (number of replica experiments) was determined based on previous experience. No statistical method was used to predetermine sample size.
Data exclusions	*Genetic data: at sample level, predefined exclusion criteria as described in the manuscript: not passing sequencing quality control thresholds and are related.<br>*siRNA screen - for primary screen following quality control of images - 13 out of 42 cell microarrays (LabTeks) were excluded from further analysis.
Replication	*Primary siRNA screen: For RNAi-based gene interaction screening, each of the five cell microarrays (LabTeks) was assayed in 7-10 independent biological replicas.<br>*Validation siRNA screen: each validated GI was assayed with 3-4 independent biological replicates; in a single replica - each siRNA treatment was represented by 100 microscope images.
Randomization	*For Genetic data - randomization was not applicable to this study. The study is observational.<br>*For siRNA screen - no randomization was performed. The study is observational.
Blinding	*For Genetic data - this study is observational, using coded de-identified data. Blinding was not applicable to this study.<br>*For siRNA screen - data collection and analysis were not performed blind.

Reporting for specific materials, systems and methods

We require information from authors about some types of materials, experimental systems and methods used in many studies. Here, indicate whether each material, system or method listed is relevant to your study. If you are not sure if a list item applies to your research, read the appropriate section before selecting a response.

Materials & experimental systems

n/a	Involved in the study
☐	☒ Antibodies
☐	☒ Eukaryotic cell lines
☒	☐ Palaeontology and archaeology
☒	☐ Animals and other organisms
☐	☒ Human research participants
☒	☐ Clinical data
☐	☒ Dual use research of concern

Methods

n/a	Involved in the study
☒	☐ ChIP-seq
☒	☐ Flow cytometry
☒	☐ MRI-based neuroimaging

Antibodies

Antibodies used

Rabbit polyclonal anti LDLR antibody Fitzgerald, 20R-LR002 (dilution 1:100); mouse monoclonal anti LDLR-C7 antibody Progen, 61087 (dilution 1:100); polyclonal goat anti-rabbit IgG Alexa 568, Invitrogen, A11011 (dilution 1:400); polyclonal chicken anti-mouse IgG Alexa 488 Invitrogen, A-21200 (dilution 1:400).

Validation

We validated both antibodies in immunofluorescence experiments with RNAi knockdown of LDLR in HeLa Kyoto cells

Eukaryotic cell lines

Policy information about cell lines

Cell line source(s)

HeLa-Kyoto cells are a strongly adherent HeLa isolate (gift from S. Narumiya, Kyoto University Japan)

Authentication

morphological authentication is performed as HeLa Kyoto cell line has a very typical morphological features

Mycoplasma contamination

All cell lines were tested negative for mycoplasma.

Commonly misidentified lines
(See ICLAC register)

There were no commonly misidentified cell lines used in the study

Human research participants

Policy information about studies involving human research participants

Population characteristics

All population characteristics of UK Biobank cohort are available at www.ukbiobank.ac.uk. Participants recruited (>500, 000 individuals) were aged 40-69. Participant data include health records, medication history and self-reported survey information, together with imputed genome-wide genotypes and biochemical measures (see Bycroft et al., 2018). Baseline biochemical measures including LDL cholesterol (LDLc), HDL cholesterol (HDLc), triglycerides (TG), and serum total cholesterol (TC) had been obtained in UK Biobank's purpose-built facility in Stockport as described in the UK Biobank online data showcase and protocol (www.ukbiobank.ac.uk). Demographic and other relevant phenotypic information was obtained from standard questionnaire data. Individual lipid phenotypes (LDLc, HDLc, TG and TC) were first modelled as dependent variables using linear regression models against covariates including age, sex, smoking, alcohol drinking status, BMI, lipid medication use and top ten genetic principle components. Residuals were then used as outcome variables in genetic analyses. Lipid medication use was obtained from self-reported questionnaire data (UK Biobank fields 6153 and 6177). CAD cases were recognized based on both self-reported diagnosis and Hospital Episode Statistics data in the UK Biobank with a code-based CAD definition as presented in the most recent CAD GWAS that included UK Biobank(see van der Harst et al., 2018). In total, 30,125 CAD cases were identified and the CAD analysis was conducted using logistic regression models adjusted for age, sex, smoking status, alcohol drinking status, BMI, lipid medication use and top ten genetic principle components. All phenotype data were derived from the UK Biobank basket "ukb27390" released on March 11, 2019.
For the 302, 355 UK Biobank participants of whom exome sequencing data were used - the average age at recruitment was 56.5 years, 54% were female and 95% were of European ancestry.

Recruitment

Participants (>500,000 individuals) aged 40-69 years were recruited to the UK Biobank on a voluntary basis. Informed consent was obtained for all participants. There are no biases that would impact the outcomes of this study.

Ethics oversight

The research in this manuscript has been conducted using the UK Biobank resource under application number 26041. All phenotype data were derived from the UK Biobank basket "ukb27390" released on March 11, 2019.
The protocols for UK Biobank are overseen by The UK Biobank Ethics Advisory Committee (EAC). For more information see https://www.ukbiobank.ac.uk/ethics/ and https://www.ukbiobank.ac.uk/wp-content/uploads/2011/05/EGF20082.pdf

Note that full information on the approval of the study protocol must also be provided in the manuscript.

Dual use research of concern

Policy information about dual use research of concern

Hazards

Could the accidental, deliberate or reckless misuse of agents or technologies generated in the work, or the application of information presented in the manuscript, pose a threat to:

No	Yes	
☒	☐	Public health
☒	☐	National security
☒	☐	Crops and/or livestock
☒	☐	Ecosystems
☒	☐	Any other significant area

Experiments of concern

Does the work involve any of these experiments of concern:

No	Yes	
☒	☐	Demonstrate how to render a vaccine ineffective
☒	☐	Confer resistance to therapeutically useful antibiotics or antiviral agents
☒	☐	Enhance the virulence of a pathogen or render a nonpathogen virulent
☒	☐	Increase transmissibility of a pathogen
☒	☐	Alter the host range of a pathogen
☒	☐	Enable evasion of diagnostic/detection modalities
☒	☐	Enable the weaponization of a biological agent or toxin
☒	☐	Any other potentially harmful combination of experiments and agents
